# Draft Genome Sequences of the Alga-Degrading Bacteria *Aeromonas hydrophila* Strain AD9 and *Pseudomonas pseudoalcaligenes* Strain AD6

**DOI:** 10.1128/genomeA.00709-14

**Published:** 2014-07-17

**Authors:** Eric M. Lenneman, Brett M. Barney

**Affiliations:** Department of Bioproducts and Biosystems Engineering and Biotechnology Institute, University of Minnesota, St. Paul, Minnesota, USA

## Abstract

*Aeromonas hydrophila* AD9 and *Pseudomonas pseudoalcaligenes* AD6 have been linked to algal cell degradation. Here we report the draft genomes of *A. hydrophila* AD9 and *P. pseudoalcaligenes* AD6 for the investigation of causative agents for algal cell degradation.

## GENOME ANNOUNCEMENT

The production of biofuels has become an important issue in recent years due to the depletion of various sources of fossil fuels and increased costs of those fuels. Lipid production by microalgae is one proposed alternative source for the production of next-generation biofuels. However, the cost of extracting lipids from algae is often prohibitive to the economic viability of the algal biofuels venture (1–3). *Aeromonas hydrophila* strain AD9 was previously demonstrated to improve lipid extraction with a mild solvent by 12-fold from the green alga *Dunaliella tertiolecta* when algal cells were first pretreated with this bacterium and then extracted with a mild solvent treatment, indicating a strong potential for lowering the costs of a common bioprocessing barrier (4). *Pseudomonas pseudoalcaligenes* strain AD6 was shown to improve lipid extraction from the green alga *Neochloris oleoabundans* in addition to *D. tertiolecta* (4). Several additional strains of *Pseudomonas* have been identified and reported as algicidal for various strains of harmful bloom-forming cyanobacteria or algae (5–7). To further understand the means by which *A. hydrophila* AD9 and *P. pseudoalcaligenes* AD6 degrade algal cells to improve lipid recovery, draft genomes for these strains were determined and annotated.

The sequencing of *A. hydrophila* AD9 and *P. pseudoalcaligenes* AD6 was performed using Illumina technology and a standard protocol for paired-end read Illumina Hi-Seq 2000 (Illumina, San Diego, CA). For *A. hydrophila* AD9, 65,533,033 reads were obtained, containing 6,618,836,333 bases, which resulted in 1,334-fold coverage. The 221 contigs were constructed using Velvet 1.0 (8), resulting in a genome size of 4.91 Mb and a G+C content of 61.3%. For *P. alcaligenes* AD6, 64,253,252 reads were obtained, containing 6,489,578,452 bases, resulting in 1,192-fold coverage. Assembly using Velvet resulted in a draft genome size of 5.39 Mb in 198 contigs with a G+C content of 62.5%. Annotation of each genome was completed using the GenBank Prokaryotic Genome Annotation Pipeline version 2.0 (http://www.ncbi.nlm.nih.gov/genome/annotation_prok).

An analysis of the genome data from *A. hydrophila* AD9 revealed a gene (accession no. EZH83771) for a potential metalloprotease with similarity to an extracellular metal protease (accession no. AB070236) isolated from *Pseudoalteromonas* sp. strain A28 that has been implicated in algalytic activity (9). Additionally, more than 30 genes for proteases or peptidases were found, including an additional extracellular peptidase (accession no. EZH79114), as well as a potential extracellular lipase of the Pla-1/cef family (accession no. EZH81869). Additional pore-forming toxins such as aerolysin (accession no. EZH82509) and hemolysin (accession no. EZH79984) were also identified. The analysis of the annotation from *P. pseudoalcaligenes* AD6 revealed more than 40 genes for proteases and peptidases and several lipases. These various proteins represent potential targets for future studies to determine the causative agent of the algicidal activity from these strains.

### Nucleotide sequence accession numbers.

The draft genome sequence of *Aeromonas hydrophila* AD9 has been deposited in DDBJ/EMBL/GenBank under the accession number JFJO00000000. The version described in this paper is JFJO01000000. The draft genome sequence of *Pseudomonas pseudoalcaligenes* AD6 has been deposited in DDBJ/EMBL/GenBank under the accession number JFJN00000000. The version described in this paper is JFJN01000000.
